# The Impact of Caregiving on the Association Between Infant Emotional Behavior and Resting State Neural Network Functional Topology

**DOI:** 10.3389/fpsyg.2018.01968

**Published:** 2018-10-15

**Authors:** Lindsay C. Hanford, Vincent J. Schmithorst, Ashok Panigrahy, Vincent Lee, Julia Ridley, Lisa Bonar, Amelia Versace, Alison E. Hipwell, Mary L. Phillips

**Affiliations:** ^1^Department of Psychiatry, Western Psychiatric Institute and Clinic, University of Pittsburgh Medical Center, University of Pittsburgh, Pittsburgh, PA, United States; ^2^Department of Pediatric Radiology, UPMC Children’s Hospital of Pittsburgh, Pittsburgh, PA, United States

**Keywords:** infant brain, resting state, neural network nodal metrics, emotion behavior, observed caregiving

## Abstract

The extent to which neural networks underlying emotional behavior in infancy serve as precursors of later behavioral and emotional problems is unclear. Even less is known about caregiving influences on these early brain-behavior relationships. To study brain-emotional behavior relationships in infants, we examined resting-state functional network metrics and infant emotional behavior in the context of early maternal caregiving. We assessed 46 3-month-old infants and their mothers from a community sample. Infants underwent functional MRI during sleep. Resting-state data were processed using graph theory techniques to examine specific nodal metrics as indicators of network functionality. Infant positive and negative emotional behaviors, and positive, negative and mental-state talk (MST) indices of maternal caregiving were coded independently from filmed interactions. Regression analyses tested associations among nodal metrics and infant emotionality, and the moderating effects of maternal behavior on these relationships. All results were FDR corrected at alpha = 0.05. While relationships between infant emotional behavior or maternal caregiving, and nodal metrics were weak, higher levels of maternal MST strengthened associations between infant positive emotionality and nodal metrics within prefrontal (*p* < 0.0001), and occipital (*p* < 0.0001) cortices more generally. Positive and negative aspects of maternal caregiving had little effect. Our findings suggest that maternal MST may play an important role in strengthening links between emotion regulation neural circuitry and early infant positive behavior. They also provide objective neural markers that could inform and monitor caregiving-based interventions designed to improve the health and well-being of vulnerable infants at-risk for behavioral and emotional problems.

## Introduction

The rapid development of the human brain in the first years of life (Knickmeyer et al., 2008; Gao et al., 2015b) is important for brain-behavior relationships that set the stage for future clinical and functional outcomes. Sensorimotor, auditory and visual networks develop first (Lin et al., 2008; Liu et al., 2008), followed by other large-scale networks important for higher-order regulatory processes (Gao et al., 2009, 2015a), including: the default mode network (DMN), implicated in self-referential processing (Amsterdam, 1972; Gusnard et al., 2001; Raichle et al., 2001; Ramenghi et al., 2009; Short et al., 2013; Alcauter et al., 2014; Ball et al., 2015); the salience network, underlying attention to personally salient stimuli (Seeley et al., 2007; Akazawa et al., 2016); and the frontoparietal executive control network, important for multiple cognitive control processes including emotional regulation (Phillips et al., 2008; Gao et al., 2016). Additionally, the normal adult pattern of inverse correlation of resting state functional connectivity between the DMN and the dorsal attention network (Corbetta and Shulman, 2002; Akazawa et al., 2016) emerges in the first year (Gao et al., 2013).

Yet, links between early alterations in neural circuitry structure and function and clinically relevant behavioral outcomes remain unclear. In normally developing infants, there is heightened neural sensitivity to environmental factors, including caregiving behaviors (Sur and Rubenstein, 2005; Roth and Sweatt, 2011). These early experiences influence the formation of synapses and dendritic projections, which alter the recruitment of specific brain regions in larger-scale networks, including those important for behavioral and emotional regulation (Huttenlocher, 1990; Kolb and Gibb, 2011). Although young infants have a rudimentary capacity to self-regulate, the expression and regulation of emotions is influenced by the provision of sensitive caregiving (Tronick and Gianino, 1986; Cirulli et al., 2003). Yet, the relationships between emotional network functioning and infant emotionality, and the impact of early caregiving on these relationships, are not well understood.

Positive and negative emotional behaviors (PE, NE) can be measured reliably within the first months of life (Worobey and Blajda, 1989; Rothbart et al., 2001; Dinehart et al., 2005). Infants displaying high levels of PE frequently smile or laugh, and experience high intensity pleasure (Tellegen, 1985; Watson et al., 1999). Some data suggest that low levels of PE in infancy precede behavioral inhibition (Park et al., 1997) and depression in childhood (Oldehinkel et al., 2004; Hayden et al., 2006; Anderson and Hope, 2008; Doucherty et al., 2010). Conversely, infants displaying high NE cry frequently and intensely in response to novelty and limitations, and are difficult to soothe (Henderson and Wachs, 2007). High NE in infancy is a relatively robust predictor of emotional problems later in childhood (Burgess et al., 2003).

Emotional regulation networks are well characterized in adults (Gray et al., 2002; Ochsner and Gross, 2005; Phillips et al., 2008), and include lateral and medial prefrontal cortical regions, specifically, anterior cingulate cortex (ACC), orbitofrontal cortex (OFC), and mediodorsal, ventrolateral, and dorsolateral prefrontal cortex (mdPFC, vlPFC, and dlPFC, respectively), within executive control and salience networks (Phillips et al., 2008; Sotres-Bayon et al., 2012; Nieh et al., 2013). Lower levels of functional connectivity between prefrontal cortical regions implicated in emotional regulation have been observed in children at risk for emotional disorders relative to healthy children (Singh et al., 2014; Manelis et al., 2015). By contrast, research examining relationships between large-scale network function and emotional behaviors in infancy is minimal. One study reported greater OFC activity to sad versus neutral vocalizations in healthy 3- to 7-month-old infants (Blasi et al., 2011), suggesting an early functional specialization for processing human auditory NE. More recently, connectivity patterns of the amygdala in early infancy have been associated with developmental changes in temperament (Graham et al., 2016).

Parental caregiving plays a critical role in the development of infant emotional behaviors and emotional regulation (Tronick and Gianino, 1986; Oldehinkel et al., 2006; Thompson and Meyer, 2007). Caregiving behaviors can be subdivided into three distinct categories; positive, negative and mental-state talk (MST) (Park et al., 1997; Sharp and Fonagy, 2008; Lipscomb et al., 2011; Meins et al., 2012). Positive aspects of caregiving, including warmth and sensitivity, predict lower NE in offspring (Park et al., 1997), whereas more negative caregiving behaviors, such as harsh and intrusive behaviors, are associated with greater infant NE (Lipscomb et al., 2011). Longitudinal studies support the *interaction* between infant emotional reactivity and maternal caregiving in shaping later clinical and functional outcomes in offspring (Kochanska, 1995; Boyce and Ellis, 2005; Oldehinkel et al., 2006; Pluess and Belsky, 2010). Thus, high NE infants, who experience negative caregiving are at highest risk for later emotional dysregulation (Morrell and Murray, 2003), while high PE may act as a buffer in negative parenting environments (Lengua et al., 2000; Prior et al., 2001; Feder et al., 2009). Another aspect of caregiving, the mother’s capacity to ‘read,’ understand and attribute mental states to her infant, is considered a *prerequisite* for sensitive caregiving (Sharp and Fonagy, 2008; Meins et al., 2012). Declarative indices of maternal MST have been linked with subsequent social–cognitive abilities in the child, including heightened emotion understanding (Doan and Wang, 2010). Our own work and that of others, has shown maternal MST to be a distinct aspect of maternal caregiving (Hipwell et al., 2016) with particular relevance for shaping emotional behaviors in the child (Meins et al., 2012; Hipwell et al., 2015).

Family environment influences on infant network function have been only briefly studied. Greater connectivity at rest between anterior and posterior regions of the DMN in 6–12 months old infants was associated with higher levels of conflict between caregivers (Graham et al., 2015). This finding parallels connectivity patterns in depressed adults and children (Sheline et al., 2010; Gaffrey et al., 2012), suggesting a link between characteristics of the early caregiving environment, NE, and alterations in network connectivity in infancy. It further suggests negative caregiving may adversely affect network development in infancy and predispose to future mental health problems (Kochanska, 1995; Boyce and Ellis, 2005; Oldehinkel et al., 2006; Pluess and Belsky, 2010). We are not aware, however, of any prior research that examined whether different types of caregiving behaviors impact neural networks, and relationships among networks and emotional behaviors, in infancy. Elucidating these relationships will be of clinical importance, as these relationships could help identify the most salient caregiving behavior targets in interventions designed to improve the health and well-being of vulnerable infants.

A robust method for establishing brain-behavior relationships is through network-based approaches, which can characterize the functional integration and segregation of large-scale networks (Supekar et al., 2009; Friston, 2011; Gao et al., 2015b). Two metrics commonly employed in such analyses are clustering coefficient (CC) and nodal efficiency (NEff). CC measures the degree to which nodes (neural regions) in a network segregate (Bullmore and Sporns, 2009; Rubinov and Sporns, 2010). Greater CC in network nodes is thought to provide more redundancy and render the network more resilient to insult to any single node (Bullmore and Sporns, 2009; Rubinov and Sporns, 2010). NEff measures the efficiency of communication of a single given node with other nodes in a network (Bullmore and Sporns, 2009; Rubinov and Sporns, 2010), and has been used to distinguish infants at-risk for future psychiatric problems including Autism Spectrum Disorder (Lewis et al., 2017). Using these network-based approaches and nodal metrics during resting state allows examination of the specific nodal contributions to the segregation and/or efficiencies of large-scale network functional topology without requiring the use of cognitive tasks, and is thus well-suited to studies in infants.

In the present study, we focused on infant network nodal metrics, infant displays of positive and negative emotional behaviors, and maternal caregiving behaviors at 3 months postpartum, as this represents a developmental window characterized by elemental forms of emotion regulation (Feldman et al., 1996; Horowitz-Kraus et al., 2017), and the appearance of early neural functional specialization for processing negative emotion (Blasi et al., 2011).

We aimed to:

(1) Identify relationships between infant emotional behaviors and nodal metrics in large-scale whole-brain networks. Based on behavioral data linking higher NE and lower PE in infancy with elevated risk for later emotional problems (Lonigan et al., 2003; Oldehinkel et al., 2004; Eisenberg et al., 2009; Doucherty et al., 2010), and observations of lower functional connectivity in large-scale networks important for emotional regulation in children at-risk for emotional problems (Singh et al., 2014; Manelis et al., 2015), we hypothesized that higher NE and lower PE in infancy would be associated with lower CC and NEff within emotion regulation networks. Specific nodes showing these relationships would include lateral and medial prefrontal cortical regions.
(2) Identify relationships between caregiving behaviors and nodal metrics in large-scale, whole-brain networks, and examine the extent to which maternal caregiving influences the relationships between infant emotional behaviors and nodal metrics in large-scale whole-brain networks. Based on findings linking positive caregiving and maternal MST with PE and self-regulation in the child (Meins et al., 2012), and findings linking higher NE and disrupted connectivity in emotional regulation networks in children at-risk for emotional problems (Singh et al., 2014; Manelis et al., 2015), we hypothesized that higher levels of positive caregiving and maternal MST would be associated with greater CC and NEff within emotion regulation networks. Specifically, we expected that these aspects of caregiving would strengthen positive relationships between infant PE, CC and NEff, and weaken inverse relationships between infant NE, CC and NEff, within these networks. By contrast, more negative caregiving would be associated with lower CC and NEff, and would strengthen inverse relationships between NE, CC and NEff in these networks.

## Materials and Methods

### Participants

Mothers (aged 19–24 years) and their 3-month-old infants were recruited from the population-based Pittsburgh Girls Study (PGS), an ongoing longitudinal study which has conducted annual assessments on 2,450 girls followed from childhood through young adulthood (Hipwell et al., 2002; Keenan et al., 2010). Pregnant or recently delivered participants were identified from PGS interview data in waves 15–17, and study eligibility was confirmed by telephone. Following written, informed consent for their own and their infant’s participation in the study, mother-infant dyads completed a research visit at the Children’s Hospital of Pittsburgh. Procedures were approved by the University of Pittsburgh Institutional Review Board.

Exclusion criteria for the mother included prenatal or concurrent substance exposure (except for marijuana use), and provision of <2 h per day of care for her infant. Exclusion criteria for infants included: <37 weeks gestation, head circumference <32 cm, birth weight <5.5 lbs, APGAR score <7 at 5 min, extended hospitalization for any reason, or any MRI contradictions.

### Demographic and Clinical Information

Mothers reported on infant age and gender. Mothers also reported on their own mood in the past week using the Edinburgh Postnatal Depression Scale (EPDS) (Cox et al., 1987), given the potential impact of maternal depression on caregiving (Wisner et al., 2002; Logsdon et al., 2006). Household poverty was indicated by maternal report of ten potential sources of public assistance (e.g., food stamps, Medicaid, and WIC). Low levels of poverty were indicated by receipt of 0–2 types of assistance, whereas moderate to high levels were indicated by three or more types.

### Behavioral Assessments

Each mother-infant dyad was filmed in face-to-face interaction. The mother was first asked to talk to her infant “in any way you want to” without the use of toys (2 min), and then to “help your child to get interested” in a specific toy (3 min). Infant and caregiving behaviors during the two episodes were coded independently, using time-sampled global ratings, by pairs of trained coders at graduate and doctoral level who were unaware of all other information about the participants.

Positive and negative infant emotional behaviors were coded on 5-point behaviorally anchored Likert scales ranging from 1 (none) to 5 (frequent, intense, prolonged displays). Mean scores across the two observation periods were computed for infant PE and NE.

Maternal behaviors were coded on five global dimensions (hostility, intrusiveness, involvement, warmth and sensitivity) scored on 4-point Likert scales ranging from 1 (none/minimal) to 4 (many/high) adapted from the Early Parenting Coding System (Shaw et al., 2006; Hipwell et al., 2015). Mean scores on each dimension were computed from observations in the two episodes. A varimax rotation principal component analysis of these mean caregiving scores resulted in two components: positive maternal caregiving (warmth, involvement, sensitivity), and negative maternal caregiving (hostility, intrusiveness) that explained 74.9% of the variance.

Frequency of maternal MST was determined from verbatim transcriptions of maternal speech during the filmed interactions. Comments about infant internal states or intentionality (e.g., Do you think that’s funny? and Do you want to go home?) were counted and reduced to 4-point scales (1 = none to 4 = five or more comments), and a mean maternal MST score from the two observation episodes was computed.

Inter-rater reliability for coded observations was examined in a random selection of 15 (26%) dyads. Intraclass correlation coefficients (ICCs) computed for absolute agreement on coded behavioral observations were high: 0.88 infant PE; 0.85 infant NE; 0.76 maternal hostility; 0.88 maternal intrusiveness; 0.91 maternal involvement; 0.85 maternal warmth; 0.70 maternal sensitivity; and 0.89 for maternal MST.

### Resting State Data Acquisition and Image Processing

Infants were scanned during natural sleep without sedation (Haney et al., 2010; Windram et al., 2012). Mothers were asked to refrain from feeding or allowing their infant to nap for a few hours before the scan appointment, then at the allotted time the infant was swaddled and fed (‘feed and bundle’ approach) in a quiet, dimly lit room and then transferred to the MR scan table when asleep. Mothers without MRI contraindications could stay with the infant in the scan room.

Two 5-min resting state functional magnetic resonance images were acquired using a gradient-echo echo-planar imaging scan sequence with the following parameters: repetition time = 2020 ms, echo time = 32 ms, field of view = 256, 32 slices, and voxel size = 4 × 4 × 4 × mm^3^.

Images were preprocessed using in-house routines, the Cincinnati Children’s Hospital Imaging Processing Software (CCHIPS) (Schmithorst et al., 2000) written in IDL (Research Systems Inc., Boulder, CO), Statistical Parametric Mapping (SPM8) software^1^, and the Brain Connectivity Toolbox (BCT) software (Rubinov and Sporns, 2010). Image preprocessing included slice time correction, motion correction, and a 2-step normalization procedure. Motion correction methods closely followed those laid out by Power and colleagues in order to avoid spurious results due to excessive motion (Power et al., 2014). Motion correction was performed using an affine transform to the best reference image within each resting state run. The reference image was selected by determining the frame within each run that best minimized an intensity-based cost function (Power et al., 2014). A two-stage procedure for spatial normalization was used: one, the reference image for each participant was normalized to an age-specific T2-weighted image (Shi et al., 2011) in MNI space, and two, all normalized reference images were then averaged to create a study-specific template, and then used as a normalization template for each participant’s specific reference image. Finally, the entire run was transformed into MNI space (using a single transformation incorporating the motion correction parameters) and intensity normalized to grand mean = 1000. Frames with an rms deviation (DVARS) of greater than 25 from the previous frame, or a total movement from a framewise displacement (FD), as estimated from the motion correction parameters, of greater than 0.2 mm from the previous frame were rejected.

Data from each of the two resting state runs were combined into a single time course. Given the rigor of the above thresholds, a threshold of at least 4 min of total data (incorporating both runs), or 3.6 min of total data (with just one run) was used to include as many participants as possible. The number of usable frames was calculated and included as a covariate of no interest. An age-specific parcellation atlas (Shi et al., 2011) was used to extract average time courses from 90 bilateral cortical regions. Each time course was band-pass filtered (0.009 Hz < *f* < 0.08 Hz), and nuisance regressors including motion correction parameters, linear and quadratic drift, and global, average white matter and CSF signal, were used. Correlation matrices (90-X-90, absolute value) were computed based on the correlations of the time courses between two regions after removal of nuisance regressors and band-pass filtering.

The correlation matrices were thresholded (yielding a binarized graph, where each possible connection between nodes has a value of either zero or one) and computed to obtain specific nodal measures. The matrices were thresholded according to differing values of cost (the ratio of the number of connections in the graph to the number of possible connections). A cost-independent analysis was used according to fixed values of cost ranging from 0.05 to 0.45 (step size 0.05). Here, subject was included as a random variable within these random slopes models, since thresholding at different cost values would be expected to have a multiplicative and not just a linear effect on the graph metrics. These models were averaged to obtain a more stable value per subject.

The formulae for CC and NEff are given below, for the binarized graphs used here. The connection between nodes *i* and *j* in the graph is designated as *G_ij_*, which has a value of zero or one. The shortest distance (or path length) between nodes *i* and *j* is designated as *D_ij_*; if the nodes are completely unconnected, the distance is infinite. The clustering coefficient of a node *i* is defined as:

$$CC_{i}=\frac{\sum_{j,k}G_{ij}G_{ik}G_{jk}}{\sum_{\begin{array}{l} j,k\\ j\neq k \end{array}}G_{ij}G_{ik}}$$

and can be interpreted as the ratio of “triangles” (e.g., nodes *i, j*, and *k* are all connected to each other) to the number of possible “triangles” (e.g., node *i* is connected to nodes *j* and *k*). The nodal efficiency of a node *i* is defined as:

$$Neff_{i}=\frac{1}{N-1}\sum_{j}\frac{1}{D_{ij}}$$

where *N* is the total number of nodes in the graph, and 1/*D_ij_* = 0 for the case where *D_ij_* is infinite (the nodes are unconnected). This metric is the average “efficiency” (reciprocal path length) of node i for communicating with all the other nodes in the graph.

### Statistical Analyses

**FIGURE 1:**
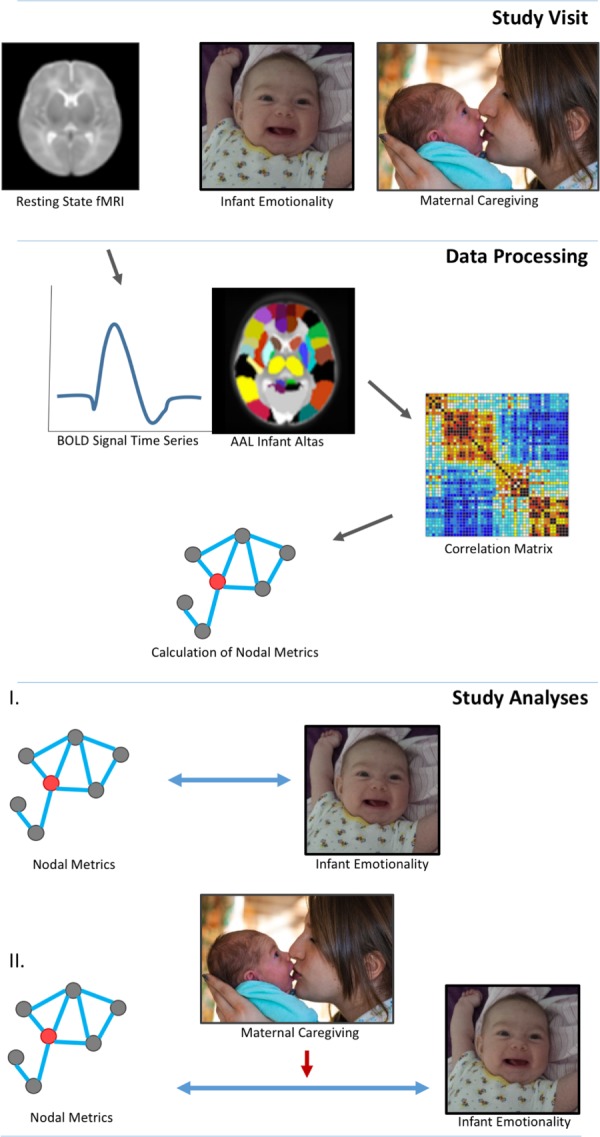
Study design and data analyses. Three-month infants were scanned during natural sleep without sedation. Mother-infant dyads were filmed in a face-to-face interaction in order to code global ratings of infant emotional behavior and maternal caregiving. Data processing involved a standard pipeline: motion correction, normalization and smoothing. Images were segmented into 90 whole brain regions of interest (ROI) based on the aal neonatal atlas. Graph theory techniques defined the interactions between nodes, specifically, clustering coefficient and nodal efficiency. Analyses determined relationships among nodal network metrics and measures of infant emotionality, and the moderating effects of maternal caregiving on these relationships. Informed written consent was obtained for the publication of mother and infant images.

See **Figure 1** for study design and statistical analysis structure.

**Aim 1:** Associations among specific nodal metrics (CC, NEff) and infant behaviors were examined in two separate regression models: where whole-brain nodal metrics were dependent variables, and either NE or PE, were independent variables.

**Aim 2:** Associations among specific nodal metrics (CC, NEff) and positive caregiving, negative caregiving or MST were examined in three regression models: where whole-brain nodal metrics were dependent variables, and either positive caregiving, negative caregiving or MST were independent variables. Next, the moderating effect of maternal caregiving on associations between infant NE and PE and specific nodal metrics was explored in six regression analyses that each included an interaction term: NE x negative caregiving, NE x positive caregiving, NE x maternal MST, PE x negative caregiving, PE x positive caregiving or PE x maternal MST, covarying for main effects of the corresponding infant and maternal caregiving measures.

In each set of analyses, infant age, gender, maternal depressed mood, use of public assistance, and number of usable frames, were covariates of no interest (**Supplementary Materials** for main effects of these covariates). Results were FDR corrected at alpha = 0.05. A mixed-effects model with an unstructured correlation matrix was used to account for the varying values of cost.

All interactions that survived multiple comparisons correction were graphed using ggplot2 (Wickham, 2016) in R^2^ to better display the relationships between infant emotional behavior, nodal metrics and maternal MST.

## Results

Resting state fMRI and observational measures of infant emotional behaviors and maternal caregiving were collected for 58 infants. Ten infants were excluded from analyses due to excessive motion, and two mothers did not complete the EPDS. Thus, 46 infants were included in total (**Table 1**). For 67% of mothers, this was their first born infant, for 27% this was their second child. For the remaining 6%, the birth order ranged from third to fifth child. The majority of mothers (76%) reported using 3–7 counts of public assistance. 20% of mothers indicated ‘probable’ postnatal depression on the EPDS (a score of >10 on the EPDS), and 12.5% had EPDS scores indicative of postnatal depression (score of >13), consistent with population norms (O’hara and Swain, 1996; Wisner et al., 2002).

**Table 1:** Demographic, clinical, behavioral, and other covariate information.

	Mean (SD)	Range
**Infant demographics**		
Age, in months	3.3 (0.8)	2–5
	***N* (%)**	
Gender, number of females	22 (48)	
Birth order, first child	32 (66)	1–5
Birth order, second child	13 (27)	1–5
**Infant emotional behaviors**		
Positive emotional behavior	2.2 (0.7)	1–5
Negative emotional behavior	1.3 (0.5)	1–2.5
**Infant covariate information**		
Number of usable frames	217.8 (58.9)	111–292
Scan length, in minutes	7.3 (2.0)	3.7–9.7
**Maternal demographics**		
	***N* (%)**	
Social assistance tally^∗^, number of moderate/high users	35 (76)	
**Maternal caregiving behaviors**		
Positive caregiving (PCA component)	-0.06 (1.11)	-4.18–1
Negative caregiving (PCA component)	-0.20 (0.92)	-1.56–3.04
Maternal mental-state talk	2.9 (0.8)	1–4
**Maternal clinical information**		
Edinburgh Postnatal Depression Scale (EPDS) score^‡^	6.1 (5.6)	0–24


*^∗^Social assistance tally was measured by counting the number of public assistance services used, these were further dichotomized into low (0–2 counts) or high (3–7 counts).*

*^‡^An EPDS score >10 was reported by 20% of mothers, indicating ‘probable’ depression, while an EPDS score of >13 was reported in 12.5%.*

Infant emotional and maternal caregiving variables are in **Table 1**. Infant negative and positive emotional behaviors were uncorrelated (*r*_s_ = -0.07, *ns*), as were positive and negative caregiving behaviors with MST (*r*_s_ = 0.29, *ns* and *r*_s_ = -0.14, *ns*, respectively).

### Network-Emotional Behavior and Network-Caregiving Relationships

No relationships between either PE or NE and CC or NEff survived FDR correction, nor did relationships between maternal positive caregiving, negative caregiving or maternal MST and CC or NEff (**Table 2**).

**Table 2:** Network nodal metric associations with positive and negative infant emotional behaviors and with maternal caregiving indices.

Region	Metric	*T* statistic	*P* statistic
**Positive emotional behavior**			
Left superior orbitofrontal cortex	CC	-2.8	<0.01
Left inferior parietal lobule	CC	-3.6	<0.001
Left caudate	NEff	2.9	<0.01
**Negative emotional behavior**			
Left paracentral lobule	CC	-3.0	<0.005
Right middle occipital gyrus	NEff	2.7	<0.01
**Positive caregiving**			
No significant results			
**Negative caregiving**			
No significant results			
**Mental-state talk**			
Left parahippocampal gyrus	CC	-2.8	<0.01
Left amygdala	CC	-2.7	<0.01
Right hippocampus	NEff	-3.3	<0.005


*CC, clustering coefficient; NEff, nodal efficiency.*

*No metrics survived FDR <0.05 correction for multiple comparisons.*

### Impact of Caregiving on Network-Emotional Behavior Relationships

There was one significant positive moderating effect of positive maternal caregiving on the relationship between PE and NEff in the left middle OFC. This PE-NEff relationship was less inverse with higher levels of positive maternal caregiving (**Table 3**). There was one significant positive moderating effect of negative maternal caregiving on the relationship between infant NE and CC in the right inferior parietal lobule. This NE-CC relationship was more positive with higher levels of negative maternal caregiving (**Table 3**).

**Table 3:** Moderating effects of positive and negative maternal caregiving on the associations between nodal metrics and positive and negative infant emotional behaviors.

Region	Metric	*T* statistic	*P* statistic
**Positive emotional behavior by positive maternal caregiving**			
Left inferior occipital gyrus	CC	-2.8	<0.01
Right angular gyrus	CC	-3.2	<0.005
Left middle orbitofrontal cortex	NEff^∗^	4.6	<0.0001
**Negative emotional behavior by positive maternal caregiving**			
Right putamen	NEff	-2.9	<0.01
Right pallidum	NEff	-2.8	<0.01
**Positive emotional behavior by negative maternal caregiving**			
Left supplementary motor area	CC	2.7	<0.01
Right inferior frontal (pars triangularis) gyrus	NEff	2.9	<0.01
Left insula	NEff	-2.8	<0.01
Left superior temporal pole	NEff	-3.1	<0.005
**Negative emotional behavior by negative maternal caregiving**			
Right inferior parietal lobule	CC^∗^	3.8	<0.0005
Left inferior parietal lobule	CC	3.2	<0.005
Left middle orbitofrontal cortex	NEff	3	<0.005


*CC, clustering coefficient; NEff, nodal efficiency.*

*^∗^Denotes results that survived FDR <0.05 correction for multiple comparisons.*

*A positive interaction denotes that the relationship between nodal metrics and infant emotionality was more positive (or less negative) with greater level of the given maternal caregiving behavior. A negative interaction denotes that the relationship between nodal metrics and infant emotionality was more negative (or less positive) with greater levels of the given maternal caregiving behavior.*

**FIGURE 2:**
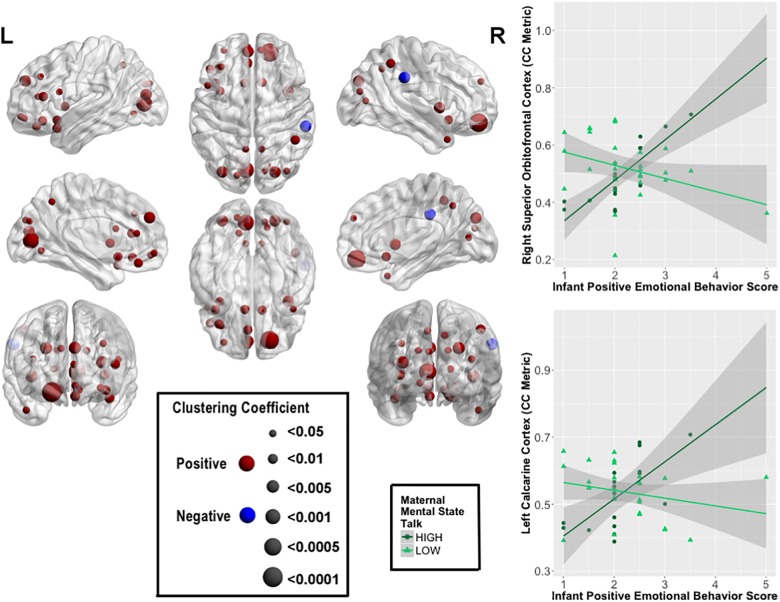
Moderating effects of maternal mental-state talk on the associations between clustering coefficient (CC) and positive infant emotional behavior (PE). The most robust moderating effects were observed within bilateral prefrontal cortical and visual processing regions. The size of the node represents the strength of the moderating effect of maternal mental-state talk on PE-CC relationships. Graphical representations of these moderating effects on the right superior OFC and left calcarine cortex are displayed. In each case, stronger positive PE-CC relationships were shown at higher levels of maternal mental-state talk; these relationships were less positive or inverse at lower levels of mental-state talk.

Several highly significant FDR-corrected moderating effects of maternal MST were observed for the relationship between PE and CC. Here, there were robust positive moderating effects of MST on PE-CC relationships in bilateral OFC, left middle and inferior frontal gyri, bilateral olfactory cortex of the OFC, bilateral medial superior prefrontal cortex, left rectus gyrus, bilateral insula, left ACC, bilateral calcarine cortex, bilateral cuneus, left lingual, bilateral superior and middle occipital gyri, right inferior parietal lobule, bilateral precuneus, and left superior and right middle temporal pole. In each case, stronger positive PE-CC relationships were shown at higher levels of maternal MST, while these relationships were weakened, becoming less positive or inverse, at lower levels of MST (**Table 4** and **Figure 2**). Negative moderating effects of maternal MST were observed for the relationship between PE and CC in the right supramarginal gyrus and between PE and NEff in the left superior temporal gyrus. Here, inverse PE-CC relationships were weakened or became positive with higher maternal MST (**Table 4**).

**Table 4:** Moderating effects of maternal mental-state talk on the associations between nodal metrics and positive and negative infant emotionality.

Region	Metric	*T* statistic	*P* statistic
**Positive emotional behavior by maternal mental-state talk**			
Right superior orbitofrontal cortex	CC^∗§^	4.7	<0.0001
Left middle frontal gyrus	CC^∗^	2.3	<0.05
Left middle orbitofrontal cortex	CC^∗^	2.3	<0.05
Left inferior frontal (opercular) gyrus	CC^∗^	2.6	<0.05
Left inferior frontal (pars triangularis) gyrus	CC^∗^	2.8	<0.01
Left inferior orbitofrontal cortex	CC^∗^	2.8	<0.01
Right olfactory cortex	CC^∗^	2.8	<0.01
Left olfactory cortex	CC^∗^	2.7	<0.01
Right medial superior frontal gyrus	CC^∗^	2.5	<0.05
Left medial superior frontal gyrus	CC^∗^	3.0	<0.005
Left medial orbitofrontal gyrus	CC^∗^	2.1	<0.05
Left rectus gyrus	CC^∗^	2.8	<0.01
Right insula	CC^∗^	3.2	<0.005
Left insula	CC^∗^	2.9	<0.01
Left anterior cingulate gyrus	CC^∗^	2.6	<0.05
Right calcarine cortex	CC^∗^	2.4	<0.05
Left calcarine cortex	CC^∗§^	3.9	<0.0005
Right cuneus	CC^∗^	2.3	<0.05
Left cuneus	CC^∗^	2.2	<0.05
Left lingual gyrus	CC^∗^	2.2	<0.05
Right superior occipital gyrus	CC^∗^	3.3	<0.005
Left superior occipital gyrus	CC^∗^	2.3	<0.05
Right middle occipital gyrus	CC^∗^	2.4	<0.05
Left middle occipital gyrus	CC^∗^	3.0	<0.005
Right inferior parietal lobule	CC^∗^	2.7	<0.01
Right supramarginal gyrus	CC^∗^	-3.0	<0.005
Right precuneus	CC^∗^	2.5	<0.05
Left precuneus	CC^∗^	2.3	<0.05
Left superior temporal pole	CC^∗^	2.7	<0.01
Right middle temporal pole	CC^∗^	2.1	<0.05
Left superior temporal gyrus	NEff^∗^	-3.7	<0.001
Right inferior temporal gyrus	NEff	-3.1	<0.005
Left inferior temporal gyrus	NEff	-3.1	<0.005
**Negative emotional behavior by maternal mental-state talk**			
Right middle temporal pole	CC	-3.1	<0.005
Right middle frontal gyrus	NEff	2.8	<0.01
Right calcarine cortex	NEff	2.9	<0.01
Left calcarine cortex	NEff	3.0	<0.005


*CC, clustering coefficient; NEff, nodal efficiency; ^∗^denotes results that survived FDR <0.05 correction for multiple comparisons. ^§^Graphical representation of these metrics available in **Figure 1**.*

*A positive interaction denotes that the relationship between nodal metrics and infant emotionality was more positive (or less negative) with greater maternal mental-state talk. A negative interaction denotes relationships between nodal metrics and infant emotionality that were more negative (or less positive) with greater maternal mental-state talk.*

The moderating effects of maternal MST did not survive FDR correction for relationships between infant NE and either CC or NEff (**Table 4**).

## Discussion

We aimed to identify large-scale neural network-emotion relationships in 3-month-old infants, and examine the impact of caregiving on these relationships. Our major findings indicate that maternal MST influenced associations between infant PE and CC predominantly in prefrontal and occipital cortical networks, where higher levels of maternal; MST were associated with stronger positive relationships between infant PE and CC within these networks. These results contrast with more limited effects of positive and negative components of caregiving on infant brain-emotional behavior relationships; and only weak relationships among nodal metrics and either maternal caregiving or infant emotional behaviors when examined without consideration of the caregiving context. These findings suggest a specific and critical role of maternal MST in shaping positive relationships between the extent of segregation, and resulting resilience, within prefrontal cortical and visual processing networks and PE in infants.

Higher levels of maternal MST were associated with stronger positive relationships, and weaker inverse relationships, between infant PE and CC among several lateral and medial prefrontal cortical regions implicated in emotional regulation (Gray et al., 2002; Ochsner and Gross, 2005). The lateral prefrontal cortex integrates emotional and cognitive information, and generates emotional responses (Gray et al., 2002). The medial prefrontal cortex, including the OFC, serves a critical role in memory retrieval, action selection, executive control and social cognition related to emotional regulation (Ochsner and Gross, 2005). Greater maternal MST was also associated with stronger positive relationships between PE and CC in bilateral insula, important for interoception and a key role in the salience network (Zaki et al., 2012; Simmons et al., 2013), and in visual cortical regions important for higher-order visual processing. Mothers’ capacity to engage in mentalizing behaviors is linked to behavioral and neural indices of empathy and reflection, and may also be a proxy for mother-infant dyadic attunement (Meins et al., 2012). We show the importance of observed maternal MST in facilitating infant positive emotion and higher levels of segregation and built-in redundancy and resilience in emotional regulation, interoception, salience and visual processing networks in early infancy.

There were negative moderating effects of maternal MST on CC and NEff in the right supramarginal gyrus and the left superior temporal cortex, respectively, where higher levels of maternal MST were associated with stronger inverse relationships between infant PE and CC and NEff. The right supramarginal gyrus is important for somatosensory processing and empathy (Reed and Caselli, 1994; Carlson, 2012; Silani et al., 2013), while the left superior temporal gyrus is critical for language, speech, and higher-level auditory processing (Bigler et al., 2007). Given the impact of maternal MST on infant brain-PE relationships described above, maternal MST may obviate the need for either the early segregation of neural networks implicated in somatosensory and empathic processing, or the integration of neural networks subserving higher-level auditory processing. Instead, this characteristic of caregiving may promote involvement of large-scale prefrontal executive control and visual networks in the shaping of infant brain-PE relationships.

There were two significant relationships relating to moderating effects of positive and negative caregiving. More positive maternal caregiving weakened the inverse relationship between PE and NEff in the left OFC, indicating that greater, rather than lower, engagement of this key emotional regulation prefrontal cortical region may help to promote positive emotion in infants in supportive caregiving environments. More negative maternal caregiving rendered the relationship between NE and CC in the right inferior parietal lobule positive. The latter region is important for directing attention to salient new or alerting environmental stimuli (Lynch et al., 1977; Singh-Curry and Husain, 2009), and thus a positive relationship between NE and CC in this region likely reflects enhanced attention to potentially alerting or threatening stimuli in infants with higher levels of NE. That this relationship was strengthened in more negative caregiving environments parallels reports showing an effect of negative caregiving in promoting greater vigilance and NE in infants (Lipscomb et al., 2011).

The absence of strong relationships among maternal caregiving behaviors and nodal metrics *per se*, or among infant emotional behaviors and nodal metrics outside the caregiving environment, indicate a specific effect of maternal caregiving in shaping early infant brain-emotional behavior relationships. Our findings also parallel those of other studies indicating effects of positive and sensitive caregiving in providing normative social, cognitive and emotional contexts for the development of self-regulatory capabilities, and greater higher-order functioning within these domains (Bick and Nelson, 2016). Studies examining the absence of positive caregiving, including maternal separation studies, and subsequent interventions, help to elucidate caregiving effects on neural networks subserving emotional regulation (Brett et al., 2015; Bick and Nelson, 2016). Here, studies of institutional care and adoption highlight the importance of caregiving on development of child neural networks (Gee et al., 2013; Bick and Nelson, 2016). The critical role of maternal caregiving in shaping infant behavior is further highlighted in rodent studies that provide links between levels of maternal attention, through extensive licking and grooming behaviors and typical rat pup development, and the development of anxiety-like behaviors in rat pups who are ignored by their mothers (Meaney, 2001). Moreover, cross-fostering studies show these latter behavioral changes can be reversed by reintroducing pups to highly attentive foster mothers (Francis et al., 1999; Weaver et al., 2004). Non-human primate studies demonstrate that social behaviors and amygdala gene expression are directly impacted by quality of maternal care (Sabatini et al., 2007). While these studies highlight the sensitivity of the early developmental period in humans, non-human primates and rodents to effects of caregiving on emotional behaviors and neural network development, our findings are the first to show a specific impact of caregiving on shaping critical neural network-emotion relationships in human infants.

The study has some limitations. The cross-sectional nature of analyses does not provide information on the direction of relationships between maternal caregiving, infant emotion and nodal metrics. Given that this is the first study to explore any relationships among these variables, however, identifying associations is a logical first step. The relatively small sample size limited our ability to examine the influence of additional environmental factors (e.g., maternal psychiatric history) on relationships among neural measures and emotional behavior. Similarly, our sample was predominantly of low SES, which limited our ability to generalize to other populations. Low SES samples are often under-represented in research, however, and thus inclusion of such samples in future infant studies will allow examination of a greater range of emotional developmental outcomes. We did not include birth order as a covariate. This can be an additional focus of study in future research. Longitudinal studies can establish the temporal ordering of associations among infant behavior, brain functional topology and caregiving within a larger sample.

We are the first to show that maternal MST, a key component of parenting linked to emotional behavior in offspring, influences the association between infant positive emotion and functional topology in prefrontal cortical and visual processing networks. Moreover, these associations were weak in the absence of moderating effects of any caregiving components. These findings thus highlight the importance of MST, above positive or negative aspects of caregiving, on development of infant brain-positive emotional behavioral relationships. These findings are clinically important, as they can provide objective neural markers to monitor the effectiveness of caregiving-based interventions targeted at strengthening infant brain-positive emotion relationships, to improve the health and well-being of vulnerable infants at risk for behavioral and emotional problems.

## Ethics Statement

This study was carried out in accordance with the recommendations of the University of Pittsburgh Institutional Review Board. The protocol was approved by the University of Pittsburgh Institutional Review Board. All mothers gave written informed consent their own and their infant’s participation in the study in accordance with the Declaration of Helsinki.

## Author Contributions

MP and AH conceived and designed experiments. JR, AH, and LH performed all the experiments. LH, VS, AH, and MP analyzed the data. LH, AH, and MP wrote the manuscript with input from all authors.

## Conflict of Interest Statement

The sponsor had no role in the design and conduct of the study; collection, management, analysis, and interpretation of the data; preparation, review, or approval of the manuscript; and decision to submit the manuscript for publication.

## Footnotes

1 http://www.fil.ion.ucl.ac.uk/spm

2 https://www.r-project.org/
