# Supplementary material for: The Impact of Caregiving on the Association Between Infant Emotional Behavior and Resting State Neural Network Functional Topology
Source: Front Psychol. 2018 Oct 15;9:1968. doi: 10.3389/fpsyg.2018.01968 (PMC6196255; doi:10.3389/fpsyg.2018.01968)
Supplement: Supplementary file 1 [file Table_1.docx]

**Supplemental Materials**

**Supplementary Results**

**Additional analysis on covariates of no interest**

Additional analyses were run to examine the relationships between covariates of no interest (number of usable frames, household poverty, and maternal depression severity) and nodal metrics in large-scale whole-brain networks. In each additional analysis, infant age and infant sex were included as covariates. The same FDR corrections were applied.

One positive relationship that survived FDR correction was observed between the number of usable frames and CC of the right rolandic operculum (see Supplemental Table 1). No significant relationships were observed between the maternal social assistance variable or maternal depression severity and nodal metrics CC or NEff.

**Supplemental Tables and Figures**

**Supplemental Table 1 – Network nodal metric associations with covariates of no interest**

| **Region** | **Metric** | **T Statistic** | **P Statistic** |
| --- | --- | --- | --- |
| ***Number of Usable Frames*** | | | |
| Right Rolandic Operculum | CC* | -4.3 | <0.0001 |
| Right Middle Temporal Gyrus | CC | -3.3 | <0.005 |
| ***Social Assistance*** | | | |
| Left Fusiform Gyrus | CC | -2.8 | <0.01 |
| Right Putamen | CC | 2.8 | <0.01 |
| ***Maternal Depression Severity*** | | | |
| No significant results |  |  |  |
| CC = Clustering Coefficient, NEff= Nodal Efficiency | | | |
| *denotes results that survived FDR < 0.05 correction for multiple comparisons. | | | |
